# Surgical treatment of canine and feline descemetoceles, deep and perforated corneal ulcers with autologous buccal mucous membrane grafts

**DOI:** 10.1111/vop.12907

**Published:** 2021-06-04

**Authors:** Valentina Mezzadri, Alberto Crotti, Samanta Nardi, Giovanni Barsotti

**Affiliations:** ^1^ Oculistica Veterinaria Genova Genova Italy; ^2^ Department of Veterinary Science University of Pisa Pisa Italy

**Keywords:** canine, corneal perforation, corneal ulcer, feline, labial mucosa, surgical treatment

## Abstract

**Objectives:**

To report the surgical technique and postoperative outcome of corneal repair with autologous buccal mucous membrane grafts in dogs and cats with descemetoceles, deep corneal ulcers, and perforated corneal ulcers with or without iris prolapse.

**Animal studied:**

Twelve cats (13 eyes) and fourteen dogs (14 eyes) were treated.

**Procedures:**

Grafts were harvested from the unpigmented superior labial mucosa using a biopsy punch. The corneal lesion was carefully debrided and cleaned. The graft was secured to the healthy cornea with a combination of simple interrupted and continuous 9–0 polyglycolic acid sutures. In 25/27 treated eyes, an overlying pedicle conjunctival graft was also performed. A temporary nictitating membrane flap was used in all cases. The pedicle conjunctival graft was trimmed about 10–20 days postoperatively. The median follow‐up period was 549.2 days (range 14–2691 days).

**Results:**

No surgical intra‐operative complications were observed. The ulcers healed and the integrity of the globe was restored in 24/27 treated eyes. Different grades of corneal fibrosis and/or vascularization and/or pigmentation were observed in all cases at the long‐term follow‐up. In two cases, enucleation was performed due to postoperative complications and one animal developed phthisis bulbi. A total of 22/27 treated eyes appeared to have regained effective visual function at the last clinical evaluation.

**Conclusions:**

Autologous buccal mucous membrane grafts appear to successfully manage severe corneal ulcers in dogs and cats, providing a useful and economical alternative to other corneal grafts.

## 1 INTRODUCTION

In veterinary ophthalmology, ulceration is one of the most common corneal diseases in companion animals.^1^

Superficial and uncomplicated ulcers heal rapidly, with minimal scar formation. In most cases, only medical therapy is required, consisting of topical antibiotics, hyaluronic acid, and cycloplegic agents.^2^ Ulcerative keratitis that extends more deeply into the corneal stroma usually involves a secondary microbial infection which initiates stromal destruction.^2^ Both matrix metalloproteinases and dysregulated endogenous proteinases can lead to the degradation of the corneal stroma, causing corneal melting. In these cases, aggressive medical therapy consisting of topical and systemic antibacterial medication, topical, or systemic matrix metalloproteinase inhibitors, and cycloplegics are indicated, unless the integrity of the globe is at risk.^3^ In a significant number of cases, despite medical treatment, corneal melting leads to progressive ulceration and even perforation.^4^

The successful use of cross‐linking of corneal collagen (CXL) as an adjunctive therapy for corneal melting keratitis was recently described in veterinary medicine.^5^, ^6^, ^7^ This procedure increases the corneal resistance to enzymatic and mechanical constraints by creating new covalent links, and it reduces the microbial burden.^8^, ^9^

In the management of corneal ulcers, to prevent possible vision loss, surgery is indicated where there is a risk of corneal perforation.^10^ Severe ulcerative corneal lesions can be surgically treated using several materials which can be divided in four groups: homologous tissue,^11^, ^12^, ^13^, ^14^, ^15^, ^16^, ^17^, ^18^ heterologous tissue,^17^, ^19^, ^20^, ^21^, ^22^, ^23^, ^24^ acellular biomaterial,^22^, ^25^, ^26^, ^27^, ^28^, ^29^, ^30^ and synthetic material.^31^, ^32^, ^33^ A combination of different techniques has also been described.^34^, ^35^, ^36^

Some of these materials require specialized instrumentation, are expensive, and are difficult to obtain or store. Moreover, some can only be used for small, superficial corneal defects and others prevent corneal evaluation during the follow‐up period.

The autologous buccal mucosa is an abundant tissue, which is relatively easy to harvest, can be used fresh, and is always available and thus has no need for storage. The buccal mucous membrane has been used in veterinary ophthalmology for eyelid defect reconstruction,^37^, ^38^ third eyelid replacement^39^ or reconstruction,^40^ keratoconjunctivitis sicca treatment^41^ and in one case of corneal perforation.^42^ In the case of deep or penetrating corneal lesions, with or without iris prolapse, an autologous buccal mucosa can be used as a tectonic graft.

The purpose of this retrospective case series study was to evaluate the efficacy, outcomes, and complications of an autologous buccal mucous membrane graft (ABMMGS) applied to the cornea of dogs and cats affected by descemetocele, deep, and perforated corneal ulcers.

## 2 MATERIALS AND METHODS

For this retrospective case series study, the medical records of dogs and cats referred for corneal ulcers and that had undergone surgery during a nine‐year period were reviewed. The criteria for inclusion were as follows: presentation with descemetocele, deep stromal ulcers, or perforated corneal ulcers, with or without iris prolapse; surgical treatment of lesions with ABMMG; and at least two follow‐up visits in the 30 days following surgery.

Data collected from each animal patient included breed, age, gender, affected eye, depth of the corneal defect, concurrent ocular and/or systemic diseases, type of surgery performed, postoperative treatments, visual function, and fundus oculi evaluation prior to surgery and at the last follow‐up.

Preoperatively all animal patients underwent ophthalmic examination in the affected eye consisting of neuro‐ophthalmic assessment (palpebral and corneal reflex, menace response, direct and indirect pupillary light reflexes and dazzle reflex), and slit‐lamp biomicroscopy (Kowa SL‐15, Kowa Company). Schirmer tear test I (STT I) (Schirmer tear test, Eickemeyer), applanation (Tono‐pen Vet, Ametek Inc and Reichert Inc) or rebound tonometry (Tono‐vet, Icare) and indirect ophthalmoscopic examination (Omega 500, Heine Optotechnik) were performed, when practicable. A fluorescein test (Ochrex, Dioptrix) was always performed except when evident corneal perforations were observed. In cases of suspected perforation, a Seidel test was performed to detect the leakage of aqueous humor through the cornea. In the fellow eye, a complete ophthalmological examination was always performed.

Visual impairment was assessed by complete neuro‐ophthalmic examination. On the basis of the results, visual function was classified as absent, uncertain, or present.

### 2.1 Surgical procedure

Different premedication drugs were injected intramuscularly (IM) or intravenously (IV) prior to anesthesia induction by IV administration of propofol (Propovet, Zoetis Italia; 2–3 mg/kg) and maintenance with inhaled 2% isoflurane (Isoflo, Zoetis Italia) and 100% oxygen, following endotracheal intubation. In all cases, surgery was performed with the aid of an operating microscope (Shin‐Nippon OP‐2, Rexxam). The corneal surface and conjunctival sac were washed with 1:50 povidone iodine solution and sterile 0.9% saline. In cases of corneal perforation, only sterile saline was used. Any necrotic corneal tissue was resected with corneal scissors, and fibrin protruding through the perforation site was trimmed.

**FIGURE 1:**
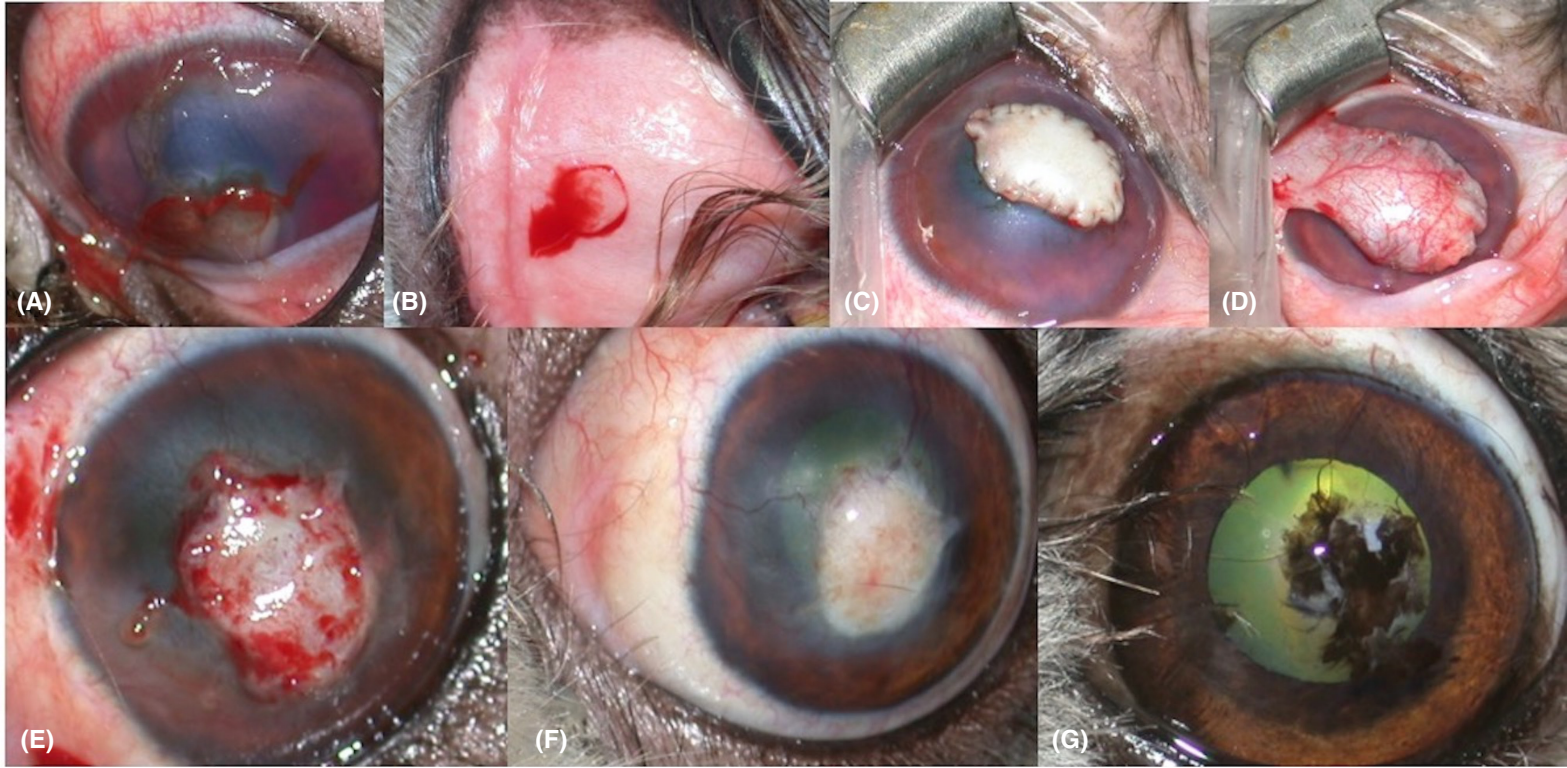
Surgical management and clinical outcome of canine case number 2. (A) Clinical appearance of left eye at presentation: corneal perforation, with iris prolapse, hyphema, and keratomalacia are present. (B) The ABMMG was obtained, using a dermal biopsy punch, from the superior labial mucosa. (C) Intraoperative photograph after suturing the buccal graft over the cornea by a simple interrupted suture with 9–0 polyglycolic acid. (D) ABMMG cover by a conjunctival pedicle graft (E) pedicle graft reshaped 15 days after surgery with topical administration of topical oxybuprocaine (F) Corneal repair after 4 months: diffuse corneal edema and vascularization over the patch were present (G) Clinical appearance after 6 years and 11 months: vascularization still present together with pigmentation of the ABMMG

A viscoelastic solution (D‐Rhexx, Dioptrix) was used to re‐inflate the anterior chamber if it had collapsed or the iris had prolapsed in the perforation site. The diameter of the corneal lesion was measured with a Castroviejo caliper. The buccal graft was harvested by a second operator from the unpigmented labial mucosa using a basic surgical set (Figure 1B). The area was prepared using a 1:10 solution of iodine povidone, and the graft was taken using a dermal biopsy punch with a 4–10 mm caliber. The labial wound was not sutured and was left to heal by second intention. Using an ophthalmic surgery set, the buccal graft was then applied over the cornea with the epithelial side up (inlay technique) to enable the epithelial cells to migrate over the graft. The graft was then sutured to the healthy tissue with 9–0 polyglycolic acid (Surgicryl, SMI,Belgium; Figure 1C).

Four cardinal sutures were placed followed by simple interrupted or continuous sutures. In 24/27 treated eyes, an overlying conjunctival pedicle graft was also performed in order to increase and improve the blood supply, using the same suture material (Figure 1D). In all the animals, a temporary nictitating membrane flap was placed using a 3‐0/5‐0 nylon suture (Daclon, SMI) depending on the animal's size. In the one case presenting bilateral corneal ulcers, the nictitating membrane fixation was performed only in the most affected eye.

### 2.2 Medical treatments

Postoperative treatments consisted of 10 mg/kg doxycycline (Ronaxan, Merial Italia) administered orally once daily for 7–14 days, 4 mg/kg carprofen (Rimadyl, Zoetis Italia) orally once daily for 7 days. All animals were discharged wearing an Elizabethan collar. At the moment of third eyelid flap removal, tobramycin ophthalmic drops (Stilbiotic, Trebifarma srl) were administered twice daily to eyes in which there were postoperative complications.

### 2.3 Follow‐up

The nictitating membrane flap was removed 1 week after surgery in all cases. The conjunctival pedicle graft, when present, was reshaped 10–20 days after surgery based on the type of initial corneal lesion and the engraftment of the graft of each clinical case (Figure 1E).

Follow‐up time ranged from 14 to 2691 days. Follow‐up examination consisted of STT 1, neuro‐ophthalmic evaluation, slit‐lamp biomicroscopy, IOP measurement, and indirect ophthalmoscopy examination.

As in the preoperative ophthalmological evaluation, visual function was assessed and classified as absent, uncertain, or present.

In cases with long‐term follow‐up (more than 6 months), a modified clinical score by^27^ was used to classify residual corneal opacities (Table 3)

## 3 RESULTS

### 3.1 Animal patients

The study population consisted of 27 eyes from 14 dogs and 12 cats. Twenty‐five animals showed unilateral corneal injuries, while only one cat presented with bilateral lesions. Table 1 for dogs and Table 2 for cats report the signalment, as well as the affected eyes, depth, extension and position of the corneal defect, concurrent ocular, and/or systemic diseases, type of surgery performed, visual impairment, and possibility of fundus oculi evaluation prior to surgery and at the last follow‐up visit.

**TABLE 1:** Data collected for canine patients

Case	Breed	Gender	Age (years)	Eye	Primary Lesion	Ulceration position and extension	Cause	Concurrent ocular abnormalities	Systemic diseases	Surgery	PO visual function	PO fundus evaluation	Follow‐up (days)	Visual function at last check	Fundus evaluation at last check	Long term complications
1	Shih‐tzu	F	4	OS	Corneal perforation with iris prolapse, melting	Lateral paracentral <25%	Unknown	Euryblepharon, medial corneal pigmentation, trichiasis, distichiasis	None	ABMMG +CPG + TEF	Uncertain	Impossible	330	Present	Possible	Lipidic keratopathy, minimal corneal opacity
2	Shih‐tzu	M	4	OS	Corneal perforation with iris prolapse, melting	Central 25%–50%	Unknown	Euryblepharon, trichiasis	None	ABMMG +CPG + TEF	Uncertain	Impossible	2539	Present	Possible	Moderate corneal opacity
3	Yorkshire Terrier	M	13	OD	Descemetocele	Central 25%–50%	Unknown	Trichiasis	Heart murmure	ABMMG +CPG + TEF	Present	Possible	499	Present	Partially possible	Moderate corneal opacity
4	Pekingese	M	2	OS	Corneal perforation, melting	Central <25%	Unknown	Euryblepharon, trichiasis	None	ABMMG +CPG + TEF	Uncertain	Impossible	65	Present	Possible	NA
5	Shih‐tzu	M	2.5	OS	Descemetocele, melting, hyphema	Central 25%–50%	Unknown	Euryblepharon, medial corneal pigmentation, trichiasis	None	ABMMG +CPG + TEF	Uncertain	Impossible	812	Present	Possible	Moderate corneal opacity
6	Shih‐tzu	M	3	OD	Corneal perforation with iris prolapse	Medial paracentral <25%	Unknown	Euryblepharon, trichiasis, distichiasis	None	ABMMG +CPG + TEF	Uncertain	Impossible	777	Present	Possible	Mild lipidic keratopathy, moderate corneal opacity
7	Beagle	F	5.5	OS	Deep stromal ulceration, melting	Central <25%	Suture notch	Bilateral KCS	None	ABMMG +CPG + TEF	Uncertain	Impossible	1055	Present	Possible	Moderate corneal opacity
8	Shih‐tzu	M	5	OD	Corneal perforation with iris prolapse, melting	Central 25%–50%	Unknown	Euryblepharon, medial corneal pigmentation, trichiasis	None	ABMMG +CPG + TEF	Uncertain	Impossible	1730	Present	Possible	Severe corneal opacity
9	English Bulldog	M	6.5	OS	Corneal perforation with iris prolapse	Central 25%–50%	Unknown	Bilateral KCS, distichiasis	None	ABMMG +CPG + TEF	Uncertain	Impossible	199	Absent	Impossible	Endophthalmitis, phthisis bulbi, severe corneal opacity
10	Shih‐tzu	M	2	OS	Corneal perforation with iris prolapse, melting	Central 50%–75%	Unknown	Euryblepharon, medial corneal pigmentation, trichiasis	None	ABMMG +CPG + TEF	Uncertain	Impossible	668	Absent	Impossible	Glaucoma, enucleation
11	Shih‐tzu	M	2	OD	Corneal perforation with iris prolapse	Medial paracentral 25%–50%	Unknown	Euryblepharon, medial corneal pigmentation, trichiasis	Chronic dermatitis	ABMMG +CPG + TEF	Uncertain	Impossible	22	Present	Partially possible	NA
12	Shih‐tzu	FS	13	OS	Corneal perforation with iris prolapse	Central 25%–50%	Unknown	Euryblepharon, medial corneal pigmentation, trichiasis, distichiasis	None	ABMMG +TEF	Uncertain	Impossible	187	Absent	Impossible	Graft rejection, endophthalmitis, enucleation
13	Shih‐tzu	M	10	OD	Corneal perforation, melting	Central 50%–75%	Unknown	Bilateral KCS, euryblepharon, trichiasis	Hearth murmure	ABMMG +CPG + TEF	Uncertain	Impossible	386	Present	Possible	Moderate corneal opacity
14	English Setter	M	11	OD	Corneal perforation with iris prolapse, melting	Central 50%–75%	Foreign body injury	None	None	ABMMG +CPG + TEF	Uncertain	Impossible	116	Present	Possible	NA

Abbreviations: ABMMG, autologous buccal mucous membrane graft; CPG, conjunctival pedicle graft; F, female; FS, sterilized female; KCS, keratoconjunctivitis sicca; M, male; NA, not applicable; OD, oculus dexter; OS, oculus sinister; PO, pre‐operative; TEF, third eyelid flap.

**TABLE 2:** Data collected for feline patients

Case	Breed	Gender	Age (years)	Eye	Primary Lesion	Ulceration position and extension	Cause	Concurrent ocular abnormalities	Systemic diseases	Surgery	PO visual function	PO fundus evaluation	Follow‐up (days)	Visual function at last check	Fundus evaluation at last check	Complication
1	DSH	FS	10	OS	Corneal perforation	Central 25%–50%	Unknown	Herpetic stromal keratitis	None	ABMMG +TEF	Uncertain	Impossible	790	Present	Impossible	Severe corneal opacity
2	DSH	FS	16	OS	Corneal perforation, melting	Central 50%–75%	Unknown	Bilateral entropion	None	ABMMG +CPG + TEF	Uncertain	Impossible	23	Present	Possible	NA
3	Persian	MC	13	OU	OS bullous keratopathy, perforation. OD bullous keratopathy	Central 25%–50%	Nigrum spontaneous extrusion	Fundus degeneration	None	OS: ABMMG +CPG + TEF OD: ABMMG +CPG	Uncertain OU	OU impossible	309	OS Absent; OD Present	OU possible	OS severe corneal opacity OD moderate corneal opacity
4	Persian	M	0,33	OD	Corneal perforation with iris prolapse, hyphema, hypopyon	Dorsonasal <25%	Cat scratch	None	None	ABMMG +CPG + TEF	Present	Impossible	14	Present	Possible	NA
5	DSH	FS	0,46	OS	Corneal perforation, melting	Lateral paracentral <25%	Cat scratch	None	None	ABMMG +CPG + TEF	Present	Possible	225	Present	Partially possible	Graft rejection, moderate corneal opacity
6	DSH	MC	16	OS	Corneal perforation, melting	Central >75%	Topical corticosteroid	None	Kidney failure	ABMMG +CPG + TEF	Uncertain	Impossible	56	Present	Partially possible	NA
7	DSH	FS	7	OS	Corneal perforation	Central 25%–50%	Cat scratch	None	None	ABMMG +CPG + TEF	Uncertain	Impossible	44	Present	Possible	NA
8	DSH	MC	5	OD	Corneal perforation with iris prolapse, hyphema	Dorsotemporal <25%	Cat scratch	None	None	ABMMG +CPG + TEF	Present	Possible	44	Present	Possible	NA
9	Persian	MC	6	OS	Corneal perforation with iris prolapse	Central 50–75%	Nigrum spontaneous extrusion	Bilateral inferomedial entropion	None	ABMMG +CPG + TEF	Uncertain	Impossible	2691	Present	Partially possible	Severe corneal opacity
10	DSH	M	1,5	OS	Corneal perforation with iris prolapse, melting	Central 25%–50%	Cat scratch, CPG rejected	Retinal detachment	None	ABMMG +TEF	Uncertain	Impossible	58	Absent	Possible	NA
11	DSH	MC	0,5	OD	Corneal perforation with iris prolapse, hyphema	Central <25%	Cat scratch	None	None	ABMMG +CPG + TEF	Uncertain	Impossible	37	Present	Possible	NA
12	DSH	FS	15	OS	Corneal perforation with iris prolapse	Central <25%	Cat scratch	None	None	ABMMG +CPG + TEF	Uncertain	impossible	622	Present	Possible	Moderate corneal opacity

ABMMG, autologous buccal mucous membrane graft; CPG, conjunctival pedicle graft; DSH, Domestic short‐haired cat; F, female; FS, sterilized female; M, male; MC, castrated male; NA, not applicable; OD, oculus dexter; OS, oculus sinister; OU, oculi uterque; PO, pre‐operative; TEF=third eyelid flap.

### 3.2 Preoperative ophthalmological findings

Corneal perforation was present in 22 eyes (11 dogs and 11 cats) of which 16 also showed iris prolapse (10 dogs and 6 cats), two dogs showed descemetocele and one a deep stromal wound; concurrent corneal melting was present in 13 eyes (9 dogs and 4 cats). The cat with the bilateral corneal lesions showed bullous keratopathy with perforation of the left eye.

On the basis of the visual assessment results, only 4 eyes (3 cats and 1 dog) showed the unequivocal presence of visual function prior to surgery. In 23 eyes, visual function was judged to be uncertain because the menace response was negative (21/23 eyes; 91.3%) or uncertain (2/23 eyes; 8.7%), while dazzle reflex in the affected eye and consensual pupillary reflex in the fellow eye were present.

Indirect ophthalmoscopic examination of the affected eyes was only possible in three cases (two cats and one dog), and no fundus abnormalities were observed.

### 3.3 Short‐term follow‐up: postoperative clinical findings up to 30 days

All surgeries were performed without complications. At the first follow‐up visit, 5–7 days after surgery, the third eyelid flap was removed in all animal patients and no complications were observed in any of the subjects.

The conjunctival pedicle graft, performed in 24/27 eyes (88.9%), was reshaped 10–20 days after surgery, depending on the complexity of the clinical case, with topical anesthesia except for two cats in which an aggressive temperament required sedation.

One dog, an English Bulldog (case 9), developed a severe endophthalmitis 23 days after surgery which led to secondary phthisis bulbi (Figure 3B). All other animal patients showed no complications at the short‐term follow‐up.

### 3.4 Long‐term follow‐up: postoperative clinical findings

Sixteen animals (17 eyes) had follow‐ups for approximately six months post‐surgery, 11 of which (11 eyes) were reassessed after more than 1 year.

**FIGURE 2:**
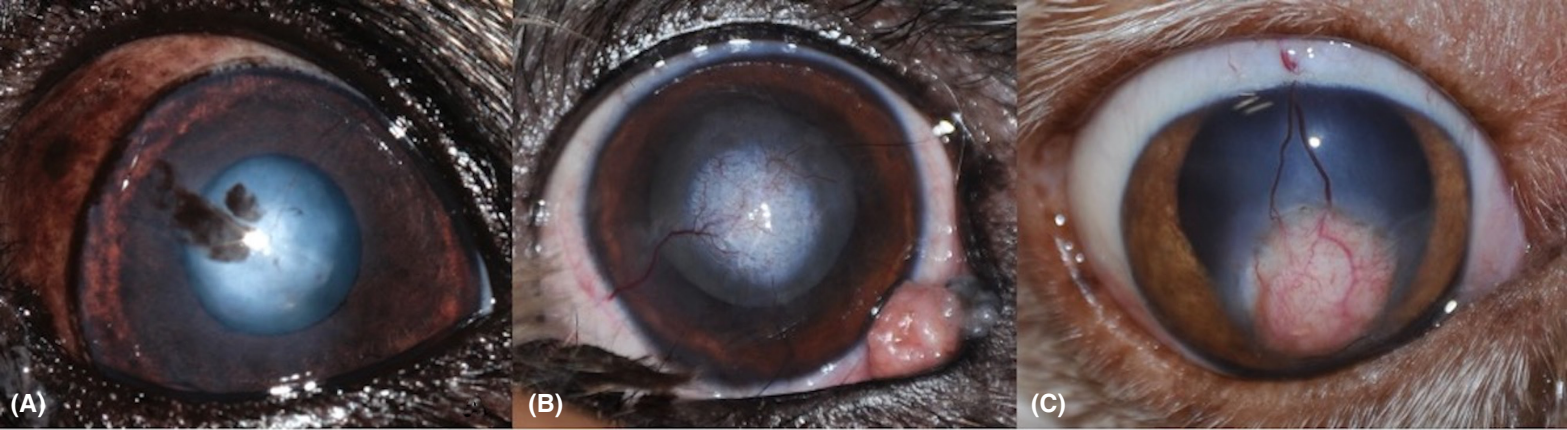
Residual corneal opacity classified as: (A) Minimal: clear visualization of the anterior chamber and posterior segment through the graft is possible. (B) Moderate: visualization of the anterior chamber and posterior segment through the graft is possible, but difficult. (C) Severe: anterior chamber and posterior segment cannot be visualized through the graft

At the last ophthalmic follow‐up visit (ranging from 187 to 2691 days), 11/17 eyes (64.8%) showed visual function, in 3/17 eyes (17.6%) visual function was judged as absent, and in 3/17 eyes (17.6%) visual impairment was considered uncertain. The fundus was completely observable in 10/17 eyes (58.9%), while in 3/17 eyes (17.6%), fundoscopy was only partially possible. Due to the degree of corneal opacity and intraocular post‐surgical sequelae, no part of the fundus was observable via ophthalmoscopy in 4/17 eyes (23.5%). Minimal opacity (grade 1; Figure 2A) was observed in 1/17 eyes (5.9%), moderate opacity (grade 2; Figure 2B) in 9/17 eyes (52.9%), and severe opacity (grade 3; Figure 2C) in 7/17 eyes (41.2%; Table 3).

**TABLE 3:** grade of residual corneal opacities

Grade	Classification	Description	Number of dogs	Number of cats
Grade 1	Minimal	Minimal stromal opacity and/or pigmentation and/or vascularization. Clear visualization of the anterior chamber and posterior segment through the graft is possible (Figure 2A)	1	0
Grade 2	Moderate	Moderate stromal opacity and/or pigmentation and/or vascularization. Visualization of the anterior chamber and posterior segment through the graft is possible, but difficult (Figure 2B)	6	3
Grade 3	Severe	Severe stromal opacity and/or pigmentation and/or vascularization. Anterior chamber and posterior segment cannot be visualized through the graft (Figure 2C)	4	3

In two cases, one dog (case 12) and one cat (case 5), graft rejection was observed after 85 days and 70 days post‐surgery, respectively. The cat underwent a second operation, and a pedicle flap was performed with a positive outcome. The dog developed an endophthalmitis which was not controllable with medical drugs, and thus, the eye was enucleated.

One Shih‐tzu (case 10) developed glaucoma secondary to an extensive anterior synechia 10 months after surgery. The pharmacological control of IOP was impossible, and the eye was thus enucleated.

**FIGURE 3:**
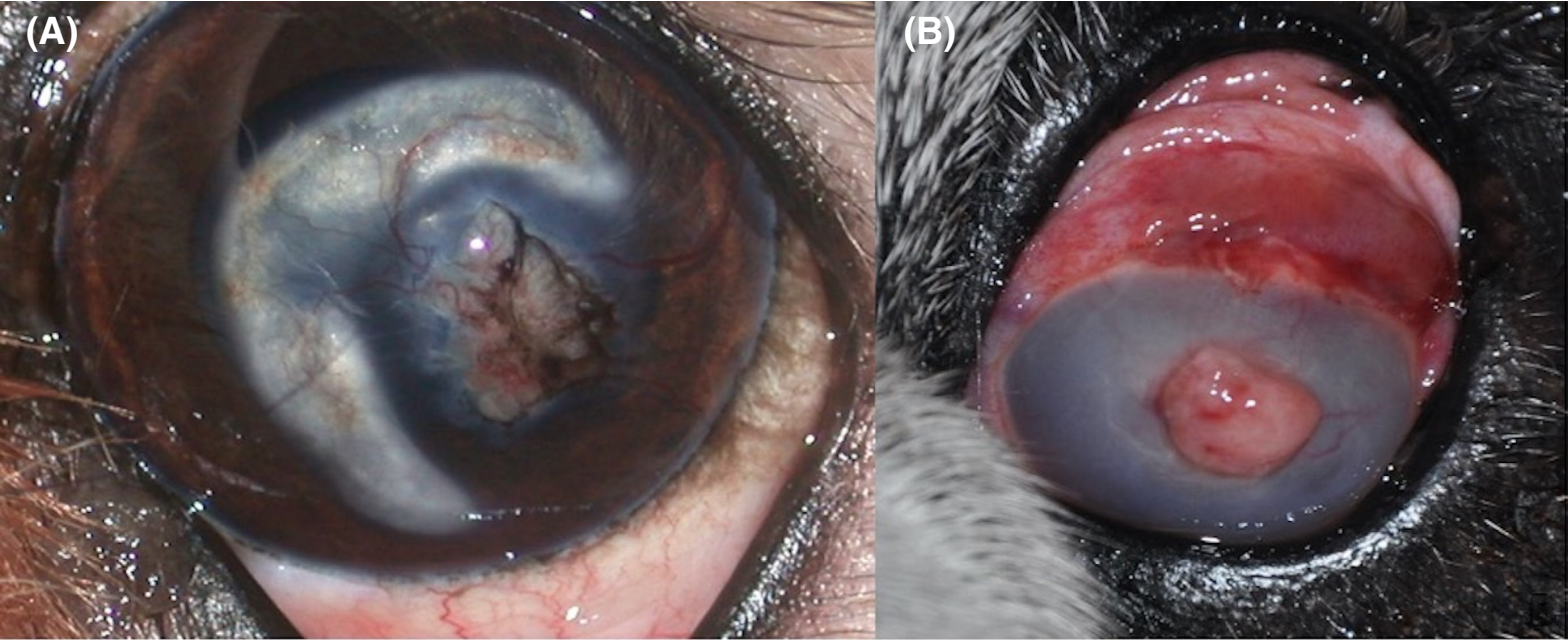
Long‐term complications (A) Canine case number 1: diffuse lipidic keratopathy (B) Canine case number 9: subconjunctival hemorrhages, endophthalmitis, phthisis bulbi, and severe corneal opacity

Two Shih‐tzus, case 1 (Figure 3A) and case 6, developed lipidic keratopathy which was evident at days 52 and 118 of the follow‐up examinations, respectively. The lipidic keratopathy was treated with a keratectomy 10 months after graft surgery in the most severe case (case 1).

## 4 DISCUSSION

Deep corneal ulcers, corneal perforations, and descemetoceles are frequently presented in veterinary ophthalmology practice, and their prompt treatment is required to prevent vision loss and ensure the anatomical preservation of the globe.^10^

Brachycephalic breeds are most commonly affected by corneal ulceration, both in cats and dogs, due to various morphological characteristics such as: corneal exposure, increased eyelid fissure, reduction in corneal sensitivity, multiple palpebral abnormalities, and nasal folds.^43^

In our study, brachycephalic canine breeds were overrepresented (11/14). In most cases, it was not possible to determine the primary cause of corneal injury, based on medical history or on ophthalmic examination. The only exceptions were the Beagle, which showed a notch suture behind the third eyelid due to a previous pocket surgery, and the English Setter in which a foreign body trauma, which occurred during a hunting incident, was reported by the owner. However, in all the other animals, there were concomitant ocular abnormalities which potentially predisposed them to corneal ulceration.

In our feline population, domestic shorthair cats were the most represented breed (9/12). In these cases, ulcerations were mostly secondary to traumatic injuries (cat scratch 6/9), while in the three Persian cats, the most common cause of presentation was the spontaneous extrusion of a corneal sequestrum (2/3).

Many different techniques have been described for deep corneal ulceration, corneal perforation, and descemetocele treatment, both in dogs and cats. These procedures can be classified into four groups of surgical techniques involving:

- homologous tissue (conjunctival graft and conjunctival island, corneo‐scleral or corneo‐conjunctival transposition, autologous corneal grafts, lamellar, and penetrating transplants using fresh or frozen corneal tissue);
- heterologous tissue (lamellar and penetrating transplants using fresh or frozen corneal tissue, equine amniotic membrane, equine pericardium, porcine amniotic membrane, equine renal capsule, bovine amniotic membrane, and human amniotic membrane);
- acellular biomaterial (porcine small intestinal submucosa, porcine urinary bladder, bovine pericardium, human amniotic membrane, and acellular corneal stroma);
- synthetic material (expanded polytetrafluoroethylene and cyanoacrylate tissue adhesive).

The conjunctival graft is the most common technique used for the surgical resolution of corneal ulcers. Several modifications of the technique were initially described in 1950,^44^ which differed above all in terms of the location and extension of the ulceration. Conjunctival grafts are easy to perform, the homologous tissue prevents the risk of rejection, provides corneal support, fibrovascular tissue to fill the corneal defects, and a blood supply.^12^ The most common complication in any type of conjunctival grafting procedure is the dehiscence of the graft and different degrees of vision impairment depending on residual corneal fibrosis. In addition, a conjunctival graft does not provide good tectonic support especially in the case of corneal perforation or in extensive descemetocele.^12^, ^16^, ^35^

The first cases in our series (cat numbers 1 and 10, dog number 12) were treated with a buccal graft alone. Although no complications were observed in the two cats, the dog had a graft rejection. We hypothesized that the dehiscence with the consequent expulsion of the graft in this case was due to the lack of blood supply. In the subsequent cases, we therefore decided to perform an additional conjunctival pedicle graft to enable vascularization of the labial mucosa graft.

Corneo‐conjunctival transposition involves mild‐to‐deep keratectomy followed by the transposition of a pedicle from a healthy cornea and its attached bulbar conjunctiva into the keratectomy site.^15^, ^16^ This technique has several advantages: the use of homologous tissue; it provides immediate vascular supply; the transposition of a clear cornea, and less central or axial corneal scarring compared to conjunctival grafting and good tectonic support.^15^, ^16^ The most common limitations are injury of the healthy cornea and the fact that it cannot be performed in extensive lesions.^1^, ^2^ Recently, a bidirectional corneo‐conjunctival transposition for the treatment of an extensive corneal sequestrum in a single cat was described.^45^ Further studies are needed to verify the applicability of this innovative technique in the surgical resolution of large perforations, with or without iris prolapse, especially in the presence of keratomalacia.

Lamellar and penetrating transplants using fresh or frozen homologous or heterologous corneal tissue are suitable for deep wounds or in very extended lesions. Due to the high level of tectonic support, these techniques are also useful in managing descemetocele or corneal perforation. Fresh cornea is difficult to harvest,^17^, ^18^ and when frozen it requires a suitable donor and freezer for storage. The major disadvantage of these techniques is the high graft rejection rate and the inflammatory response of the eye to intraocular surgery.^18^ Furthermore, considering long postoperative immunosuppressive therapy, they are not recommended in elderly patients and for melting ulcers.^18^

The amniotic membrane is frequently used in veterinary ophthalmology thanks to its anti‐inflammatory and antimicrobial effects, the inhibition of proteinases and proliferation, and the differentiation of fibroblasts.^21^, ^22^, ^23^ Until a few years ago, the technique involved the use of fresh amniotic membranes, which were difficult to find. Today there are commercially available products in cryopreserved or lyophilizate form.^22^ However, these products are very expensive and require the application of multilayers in the case of deep corneal ulcers and descemetoceles, and their use remains contraindicated in the case of perforations.^18^

Various kinds of acellular biomaterials are now on the market which are easy to acquire and store. In addition, their acellularity is associated with a lower immune response and therefore limited graft rejection,^22^, ^30^ and there is a good final transparency of the cornea.^29^, ^30^ However, their use alone is not recommended in the case of a perforation or very deep ulceration,^36^ and some authors suggest that their collagenous nature could be potentially used as a substrate for the action of collagenases.^22^

In our study, most cases presented severe lesions such as descemetoceles or corneal perforation with or without iris prolapse (25/27 eyes), and concomitant corneal melting was present in 13/27 eyes. These features prevented the use of some of the above‐mentioned techniques and, in other cases, limited finances led to less expensive procedures and with a low risk of rejection to prevent further surgery.

The advantages of the buccal mucosa are a complete and readily available tissue. It is also easy to obtain and use, and as it is a homologous tissue, it has a poor immune response with low rejection rates. In addition, the labial mucosal graft provides a strong tectonic support and thus worked better in corneal lesions with a bulging ulceration bed as in the majority of our cases presented. Based on our previous experience (unpublished data), the conjunctival pedicle graft performed alone does not work with this kind of corneal ulceration.

Although 85.1% of eyes showed moderate‐to‐severe corneal scaring, a fundus evaluation was only possible in 14.8% of cases at the final check‐up. Moreover, at the first presentation, an indirect ophthalmoscopy evaluation was not possible in 88.2% of eyes. Only 18.5% of patients showed a negative menace response at the final check‐up against 74.1% at the first presentation.

The limitations of this study include the retrospective design and the lack of a comparative group. Incomplete data collection especially regarded the long‐term follow‐up which was only available in 16/26 animal patients.

We believe that ABMMGs represent a possible alternative to other surgical techniques in large ulcerative corneal lesions with perforation, where a strong tectonic support is required. The technique restores the globe and, despite secondary corneal fibrosis, visual function seems to be maintained in most cases. Finally, ABMMGs are cheaper than other techniques and represent the best choice when the owner does not have large financial resources.
